# Improving Adenovirus Based Gene Transfer: Strategies to Accomplish Immune Evasion

**DOI:** 10.3390/v2092013

**Published:** 2010-09-24

**Authors:** Sergey S. Seregin, Andrea Amalfitano

**Affiliations:** 1 Department of Microbiology and Molecular Genetics, Michigan State University, East Lansing, MI 48824, USA; E-Mail: seregin@msu.edu; 2 Department of Pediatrics, Michigan State University, East Lansing, MI 48824, USA

**Keywords:** recombinant adenovirus, innate immunity, adaptive immunity, neutralizing antibodies, cellular responses, humoral responses

## Abstract

Adenovirus (Ad) based gene transfer vectors continue to be the platform of choice for an increasing number of clinical trials worldwide. In fact, within the last five years, the number of clinical trials that utilize Ad based vectors has doubled, indicating growing enthusiasm for the numerous positive characteristics of this gene transfer platform. For example, Ad vectors can be easily and relatively inexpensively produced to high titers in a cGMP compliant manner, can be stably stored and transported, and have a broad applicability for a wide range of clinical conditions, including both gene therapy and vaccine applications. Ad vector based gene transfer will become more useful as strategies to counteract innate and/or pre-existing adaptive immune responses to Ads are developed and confirmed to be efficacious. The approaches attempting to overcome these limitations can be divided into two broad categories: pre-emptive immune modulation of the host, and selective modification of the Ad vector itself. The first category of methods includes the use of immunosuppressive drugs or specific compounds to block important immune pathways, which are known to be induced by Ads. The second category comprises several innovative strategies inclusive of: (1) Ad-capsid-display of specific inhibitors or ligands; (2) covalent modifications of the entire Ad vector capsid moiety; (3) the use of tissue specific promoters and local administration routes; (4) the use of genome modified Ads; and (5) the development of chimeric or alternative serotype Ads. This review article will focus on both the promise and the limitations of each of these immune evasion strategies, and in the process delineate future directions in developing safer and more efficacious Ad-based gene transfer strategies.

## 1. Introduction

By 2003, 160 clinical trials were initiated utilizing Adenovirus (Ad) as a vector platform. As of 2010, there are now over 387 registered Ad-based clinical trials, (with a significant fraction of these trials still open) confirming a trend that more widespread utilization of Ad-based vectors has occurred, reflected by its use in a variety of human applications [1,2]. In these trials, Ad vectors are being administered via numerous routes. However the frequent need for use of high doses of the vector (to achieve clinically relevant levels of gene transfer) increases unwanted side effects. These include vector-associated innate immune responses and lack of efficacy due to pre-existing anti-Ad immunity in the host [3–8].

In regards to the gene therapy aspect, Ad-triggered innate immune responses are clinically relevant, unfortunately as primarily noted in the Ornithine transcarbamylase gene transfer tragedy [9]. In that trial, the tragic death of a patient coincided with induction of a “cytokine storm” – a massive release of pro-inflammatory cytokines (including IL-6), causing a systemic inflammatory response syndrome. Although not replicated in other patients receiving similar doses of the vector (both in this trial and in other subsequent studies [9–12]), the trial highlighted the need for greater study of Ad vector induced innate immune responses.

It is now known that Ad vectors elicit multiple innate immune responses after systemic administration due to several processes: complement system activation, anaphylotoxin release, macrophage activation, cytokine/chemokine release, endothelial cell activation, generalized transcriptome dysregulation in multiple tissues, activation of macrophages and dendritic cells, mobilization of granulocyte and mast cells, and thrombocytopenia [2–8,13–19]. Any or all of these responses can be detected when Ad vectors are administered *in vivo* at high doses, and these responses can lead to mortality in small animal models and primate studies [20].

There are several mechanisms underlying Ad vector triggering of the innate immune system. Ads have been confirmed to activate multiple pattern recognition receptors (PRRs) both *in vitro* and *in vivo*, including RIG-I-like receptors and Toll-like receptors: TLR-2, TLR-4 and TLR-9 (for review see [5]). These activations ultimately result in many of the previously described Ad-triggered innate toxicities. In addition, Ad-based vectors are capable of activating the human complement system via both classical and alternative complement pathway activation. Furthermore, the lack of a functional complement system (*i.e.* utilizing C3 knockout mouse models) prevents Ad vectors from triggering several important innate and adaptive immune responses [4,6,7,21–23].

Additionally, Ad-based gene transfer approaches can be hindered due the induction and/or presence of adaptive immune responses to the virus or the transgene it encodes. These adaptive immune responses can limit the duration of transgene expression, (although this highly depends on the immunogenicity of the transgene delivered [24] or animal model/strain utilized [25]) and/or limit the ability to re-administer the vector [26]. Though it is often noted that Ad-mediated gene delivery is transient due to these responses, there are multiple examples that even first generation Ads, and certainly advanced generation Ads, can allow for long-term gene expression *in vivo*, including primate models [26–29]. For example, well over 10 years ago it was first shown (and subsequently confirmed using other genes), that long-term (greater than one year) expression of the erythropoietin (hEpo) could be achieved if homologous genes were expressed by first generation Ad based vectors in a immune tolerant host [26,30–32]. In fact this logic holds true for use of gene transfer vectors in general. For example, AAV-based vectors can allow for long-term expression of homologous genes [33], but when immunogenic heterologous genes are expressed, transient expression and induction of transgene specific humoral and cellular immune responses are again encountered [34].

In addition, the high prevalence of antibodies to endemic Ad serotypes in human populations, often exceeding 50%, (with high fraction of antibodies being classified as neutralizing) contributes to the problem of pre-existing Ad immunity. Moreover, pre-existing T cell responses to Ads can also contribute to pre-existing Ad specific adaptive immune responses. These cellular responses may be more problematic than humoral immune responses, as these cellular adaptive immune responses to Ads have been shown to recognize multiple diverse, cross-clade Ad serotypes subsequent to exposure to only a single Ad serotype [35–38].

**Figure 1.**
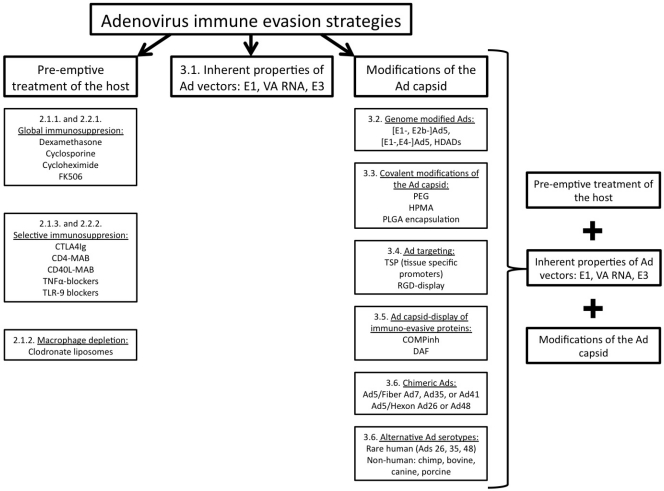
**Adenovirus immune evasion strategies.** The following scheme summarizes approaches, currently employed to minimize Ad-triggered activation of the innate and adaptive immune systems. Two main classes are (1) pre-emptive modification(s) of the host or (2) modifications of the Ad capsid.

The realization that numerous innate and adaptive immune responses can and do limit the utility of Ad based gene transfer is a sobering fact, however, it is a reality not limited to Ad-based gene transfer vectors, but to every other vector (both viral and non-viral), currently being envisioned for use as a gene transfer agent [39–43]. The widespread use of Ads has facilitated strong evidence as to the far reaching potential that gene transfer may have upon human or animal diseases, this proclivity has also fueled investigators to test and/or develop a number of innovative strategies to minimize the inflammatory responses acutely induced by Ad vectors, and/or to improve the efficacy of Ad-based gene transfer vectors over the long-term, or in the face of pre-existing Ad immunity. These approaches can be divided into two major groups as shown on Figure 1: (1) pre-emptive modification(s) of the host or (2) modifications of the Ad capsid. The promise and limitations of each of these approaches are detailed below. Finally, as has been repeatedly demonstrated in the past, the tenets established by these investigations of Ad-based gene transfer will likely be adapted to use in those studies utilizing other viral and non-viral gene transfer vector systems.

## 2. Pre-emptive treatment of the host as a strategy to allow immune evasion by Ads

### 2.1. Suppression of innate immune responses induced by Ad mediated gene transfer

#### 2.1.1. Global immunosuppression approach (innate)

The basis of this approach lies in a very simple idea: utilize currently approved non-invasive drugs to transiently suppress the immune system of the host to allow for safer and/or more efficacious gene transfer. In attempts to minimize detrimental side effects associated with prolonged immunosuppression, transient utilization of a potent, synthetic, anti-inflammatory glucocorticoid (such as dexamethasone (DEX)) [44] has been utilized to minimize the acute inflammatory responses induced by subsequent Ad administrations. For example, intraperitoneal (IP) injection of DEX for three days (5 mg/kg), one day prior to pulmonary Ad administration resulted in a significantly reduced pro-inflammatory leukocyte infiltration into the lung, and minimized pro-inflammatory cytokine release (including IL-6 and TNFα), each correlated with increased transgene (β-Gal) expression in the lung, as compared to control mice [45]. Similarly, just two doses of DEX pre-treatment (10 mg/kg, 15 and 2 hours) before systemic Ad injection completely abrogated serious, Ad-triggered innate immune responses (including thrombocytopenia, pro-inflammatory leukocyte infiltration into the liver, induction of pro-inflammatory gene expression in liver / spleen tissues, cytokine elaborations (IL-6, IL-12, MCP-1, G-CSF and other), and endothelial cell activation) as well as reduced the induction of Ad-specific humoral responses [8]. However, few studies on non-human primates have been done in this regard as our review was only able to uncover a single such study, where intramuscular (IM) pretreatment with 0.4 mg/kg DEX was used as a prophylactic to reduce Ad-induced inflammation in green monkeys [46,47].

#### 2.1.2. Macrophage depletion

Macrophages are a critically important component of the innate immune system. In particular, the liver resident macrophages known as Kupffer cells take up and degrade up to 90% of systemically administered Ad vectors [48]. Based upon this realization, the transient depletion of macrophages (by dichloromethylene bisphosphate (Cl_2_MBP) or gadolinium chloride (GdCl_3_)) appeared to be an attractive approach to minimize Ad vector clearance and, therefore, improve gene transfer outcomes. Initial studies confirmed increased persistence of Ad genomes in murine livers and lungs upon depletion of macrophages [14,49]. Infusion of liposome-encapsulated clodronate (and thereby depletion of macrophages) was also shown to enhance Ad-based hepatic gene transfer in BALB/c mice at least 4-fold [50]. It was suggested that upon Kupffer cell depletion, the rate of clearance of Ad genomes from mouse livers was reduced, thereby allowing for increased transgene (human alpha 1-antitrypsin (hAAT)) expression [50]. These results positively correlated with delayed or reduced transgene specific antibody responses [50]. Similarly, significant reductions of some of Ad-triggered systemic cytokine and chemokine levels (including IL-6 and IL-12, MCP-1 and RANTES) were noted if pre-treatment with liposome encapsulated clodronate had occurred [15,51]. Ad-triggered Erk1/2 activation in murine livers was also shown to be completely mediated by Kupffer cells, since clodronate mediated depletion completely abrogated liver Erk1/2 activation by Ads [51].

Despite the promising results in small animal models, it is still difficult to envision translation of these approaches into human clinical studies relative to systemic depletion of Kupffer cells or macrophages for facilitation of Ad mediated gene transfer. In contrast, use of these compounds in localized applications (usage of clodronated liposomes in trials for rheumatoid arthritis and other autoimmune diseases [52]) has occurred, but not in concert with Ad vector mediated gene transfer.

#### 2.1.3. Selective immunosuppression approach (innate)

Selective immunosuppressive approaches have also been tested in the context of Ad vector mediated gene transfer. For example, it has been proven that innate immune cells can sense Ad vectors via TLR9, which in turn, triggers systemic pro-inflammatory cytokine release both *in vitro* and *in vivo* [53]. TLR9 blockade with the immunomodulatory TLR9 antagonist oligonucleotide (ODN) ODN-2088 resulted in significantly reduced systemic Ad-triggered immune responses in mice, including significantly reduced plasma cytokine levels of IL-6 and IL-12 [53]. Utilization of anti-TNFα monoclonal antibodies (*i.e*.: anti-mouse TNFα IgG2a MAB) were also shown to minimize Ad-triggered innate immune responses in the pulmonary system [54]. Similarly, TNFα blockade facilitated by pretreatment with a dimeric mouse TNFR1-IgG antibody significantly reduced Ad-triggered immune responses despite systemic Ad administration. This benefit included minimized infiltration of macrophages and NK cells into the Ad-transduced liver, a result that correlated with significantly reduced induction of anti-Ad antibodies, and increased liver transgene expression [55]. This study also confirmed that the efficacy of TNFR1-IgG was similar to that of the anti-TNFα drug, Etanercept®, (human TNFR type 2 fused to human Fc region of immunoglobulin) [55]. However, treatments with TNFα blockers were continued for extended periods of time after Ad injection (for at least 24 days) [55], and may predispose to a more global immune suppression. Finally, IP injection of an Erk inhibitor (U0126), 160 mg/kg, 30 minutes prior and six hours post-Ad injection, was able to reduce some aspects of Ad-triggered inflammatory responses, including reduced activation of IP-10 [56].

Unfortunately, most of these approaches have not been tested in primate models, and, in fact, showed relatively marginal improvements in the small animal models. Additionally, use of Erk pathway inhibitors or TNFα blockers may reduce the propagation capability of oncolytic Ads (in direct tumor injection studies) [54,57].

### 2.2. Suppression of adaptive immune responses induced by Ad mediated gene transfer

#### 2.2.1. Global immunosuppression approach (adaptive)

Immunosuppression with currently approved, immune-active drugs can also improve aspects of the adaptive immune responses to Ad mediated gene transfer. For example, immunosuppression with cyclosporine A, cyclophosphamide, or FK506 have been shown to improve the efficacy of Ad-based gene transfer [58–60], most often as evidenced by decreased induction of Ad capsid neutralizing anybodies (NAB) [61] resulting in some improved efficacy upon Ad vector re-administrations [62]. Specifically, daily IM injections with cyclophosphamide (30 mg/kg) and FK506 (5 mg/kg), for 1 week prior to systemic Ad administration into rats, allowed for efficient transgene expression, even in Ad-immune animals [61]. Note, that these initial studies utilized high doses of immunosuppressive drugs for extended periods of time, often administered daily over 1–2 weeks via intravenous (IV), IM or IP injections. This is in contrast to use of DEX, a transient treatment that also reduced Ad capsid specific humoral responses, inclusive of NABs [8].

Despite heavy usage of these immunosuppressive drugs in organ transplantation and in autoimmune conditions, no clinical trials to date have specifically tested utilization of these globally immunosuppressive drug regimens during Ad mediated gene transfer. This is likely due to several reasons, including the potential risk of provoking side effects of gene transfer that might only occur in an immuno-compromised host and a lack of studies in non-human primate models. However, as greater numbers of immuno-competent patients are treated with Ad vectors, these potential risks become less problematic, and clinical trials in which the risk/benefit ratio of combining Ad mediated gene transfer with global immunosuppression will become clinically viable. This progression has already occurred at some level in use of AAV based vectors in humans. For example, use of AAV vectors in the eye was accompanied by use of transient glucocorticoid administrations (prednisone or other) [63–65], and in hemophilia B patients intravenously administered AAV vectors, induction of anti-AAV CTL responses in initial trial subjects has now warranted potential future studies proposing global immune suppression [39].

#### 2.2.2. Selective immunosuppression approach (adaptive)

In contrast to the previous approaches that utilize drugs that tend to “globally” and non-specifically immuno-suppress the host, more selective immunosuppressive approaches have been developed. For example, use of the recombinant protein CTLA4Ig (fusion of immunoglobulin with CTLA4, which binds to B7 on APCs, thereby blocking B7 / CD28 interaction and T cell activation) or anti-CD40 ligand antibodies (block CD40 / CD40L interaction, thereby blocking B cell activation) have been shown to block co-stimulatory interactions between T cells and antigen presenting cells [66,67]. These molecules (co-stimulatory antagonists) have also been utilized in clinical solid-organ transplant trials [66]. When these molecules were co-administered with Ad vectors expressing human alpha-1-antitrypsin (hAAT), they increased the duration of transgene (hAAT) expression in the host (liver: up to 180 days, lung: up to 90 days), and/or increased the efficiency of Ad vector re-administrations; the latter results positively correlated with reduced inductions of Ad-specific NAB and T cell infiltrations in the treated animals [58,68]. Similarly, pre-treatment with anti-CD40L MAB reduced the induction of Ad specific NAB titers, and allowed for more efficacious transgene expression in both mice and primates [69,70]. Specifically, pre-treatment with anti-CD40L MAB (hu5C8), at a dose of 5 mg/kg, via the IV treatment route reduced Ad-triggered cytokine elaborations (including reduced IFNγ and IL-4 production by PBMCs), results that correlated with only marginal increases in the duration of transgene expression [70], suggesting that studies in non-human primate models do not confirm the promise of these approaches.

In another approach, pre-treatment of mice with an anti-TCR MAB (H57) significantly increased the duration of transgene (β-Gal) expression [71]. Similarly, the use of anti-CD4 MABs (YTS 191) anti-CD8 MABs (YTS 169), and anti-CD11a MABs (TIB 213), in combination, allowed for prolonged transgene expression (over one month), a result that correlated with abrogated cellular and humoral immune responses to the Ad vectors in mice [72]. Rhesus macaques treated with anti-CD4 MAB (OKT4A), 10 mg/kg, IV over a 12 day period (starting a day prior to intra-bronchial Ad injection) [73] resulted in dramatically reduced pro-inflammatory leukocyte infiltrations into the primate lungs, reduced IFNγ activation in PBMCs derived from the animals, and reduced Ad specific NAB titers, all as compared to non-pre-treated Ad-injected macaques. Despite these partial decreases in Ad-triggered immune responses, no significant prolongation of transgene expression was detected [73].

Alternatively, immune stimulatory genes can be expressed directly from the Ad vector, including TLR agonists (rEA [74]), pro-inflammatory cytokines/chemokines (GM-CSF [75,76], IL-12 [77,78]), and co-stimulatory molecules (B7.1 [79], CD40L [80]). Conversely, studies have been described in which the Ad vector was specifically designed to express proteins that specifically suppress the immune system in order to improve or prolong Ad-derived transgene expression and/or improve transplantation outcomes [81]. Ad-driven expression of CTLA4Ig [81–84], anti-CD4 antibody [85], ICOSIg [83], TGFβ [84], or IL-10 have all been described. Specifically, Ad vector mediated expression of murine-CTLA4Ig significantly improved transgene (hAAT) expression from the same vector, a result that directly correlated with reduced Ad and hAAT specific humoral and cellular (T cell proliferation) immune responses [82]. Ad vector expression of anti-CD4 in *ex vivo* transduced islets improved protection of these cells against allogenic rejection in murine models [85]. Use of CTLA4Ig- and / or ICOSIg-expressing Ads, significantly reduced inflammation in a rat model of experimentally induced autoimmune myocarditis, a result that was confirmed by reductions in both T cell proliferation and IFNγ production in the Ad treated animals [83]. Importantly, Ads, expressing immuno-modulators can be administered via multiple routes, each of which can provide high plasma levels of the Ad-encoded immuno-modulators, and significant inhibition of immune responses in targeted tissues, inclusive of reduced levels of CD4 and CD8 T cell infiltrations, and/or decreased production of IL-2, IL-4, or IFNγ [86]. Whether Ad mediated expression of these immune-modulators provides greater efficacy than direct administration of these same proteins is a difficult question to answer, and has yet to be adequately addressed. However, in some instances the use of a single Ad vector to simultaneously produce various gene products may ultimately reduce the cost of such paradigms, relative to cGMP production.

## 3. Modification of the Ad genome and/or capsid as a strategy to facilitate immune evasion by Ads

### 3.1. Inherent properties of Ad vectors

The early region genes (E1-E4) of the Ad genome are the first to be transcribed and translated upon infection of a host cell, the resultant proteins expressed by these genes assist in subsequent Ad replication and packaging. The E1a (immediate early 1a) gene, facilitates transcriptional activation of all of the early genes; the E1b–encoded protein blocks apoptosis due to induction of cell cycling by the Ad E1a gene product, as well together with the E1a, E3 and E4 gene products modulate cellular transcriptional machinery to transcribe predominantly viral genes and evade host innate immune responses. Specifically, the E1B-55K and E1B-19K gene products inhibit cellular apoptosis by binding the cellular p53 protein and pro-apoptotic members of the BCL-2 family, respectively, thereby repressing their functions [87]. Most vectors for gene transfer are deleted for the E1 region of genes however, and they will not be further discussed as other chapters in this volume extensively detail use of E1a+ and/or E1b+ Ad vectors. The Ad also expresses a short, non-coding virus-associated RNA (VA RNA) that blocks IFN responses elicited by the host by binding to and blocking dsRNA-activated protein kinase R; acting as a positive regulator of Ad mRNA translation. The E3 proteins block TNFα activation and initiation of apoptosis, as well the E3-gp19K protein was specifically shown to block the transport of MHC class I molecules to the surface of the cell [87,88]. Importantly, E3-gp19K fails to inhibit transport of MHCI antigens in BALB/c mice, likely due to weak association with this (H-2D^d^) haplotype [89].

Most Ad vectors are E3 deleted, for increased cloning capacity. Furthermore, retainment of the E3 gene encoding regions within an [E1–, E3+]Ad vector would likely be minimally effectual, as the E3 gene promoters are dependent primarily upon the trans-activation capabilities of the E1 gene products. There have been studies where the E3 region (or selected genes from E3) is re-introduced into the Ad vector under appropriate control of E1 independent promoters. These studies have shown some improvement in small animal models, including reduced humoral and CD8 T cell responses to the vector, and/or long-term transgene expression [87,88,90]. Moreover, oncolytic Ads have, in some cases, the E1 regions intact and, therefore, potentially could benefit from expression of these immune evasion proteins [91].

### 3.2. Genome modified Ad vectors

The theory that removing endogenous viral genes minimizes the number of viral epitopes present for immune detection by the host has been proven in numerous studies [92–100]. Since this topic has been recently and extensively reviewed elsewhere [16,26,101], and several genome modified Ads are described in the same issue of this journal, we will not further detail these forms of immune-evading Ad vectors here.

### 3.3. Covalent modifications of the Ad capsid

One rationale for covalent modification of the Ad capsid is the possibility of shielding antigenic epitopes on the viral capsid (mainly hexon and fiber proteins [94–98]) from the hosts’ immune detection systems. This has been primarily attempted by attaching synthetic polymers to the Ad capsid inclusive of polyethylene glycol (PEG) [102], polyactic glycolic acid (PLGA) [103] or lipids [104,105].

PEG has been the most studied and widely utilized polymer in this regard, and PEGylation of Ads has been shown to significantly reduce Ad-triggered innate immune responses and associated toxicities, including the ablation of clinically relevant manifestations, such as pro-inflammatory cytokine or chemokine releases, thrombocytopenia, or pro-inflammatory leukocyte infiltrations into host (murine) tissues [105–108]. Specifically, PEGylated conventional or genome modified (i.e. HDAds) Ads have been shown to trigger significantly reduced (or in some reports completely abrogated) plasma levels of important pro-inflammatory cytokines (IL-6, IL-12, TNFα), when administered systemically, as compared to unmodified Ads [106-108]. Importantly, systemic injection of high doses (i.e. 1×10^11^ vp/mouse) of PEGylated Ad vectors allowed for prolonged transgene (β-Gal) expression in murine livers, which correlated with a significantly reduced activation of Ad-specific T cell responses (both CD8 and CD4 T cells) and blunted Ad-capsid NAB responses [106]. PEGylated Ads also allowed for sustained transgene expression in the lungs, possibly due to reduced induction of Ad NAB responses [102]. Another study combined Ad PEGylation with methylprednisone treatments (4 mg), and found that this combination completely abrogated activation of IL-6, as well resulted in reduced liver mRNA levels of several pro-inflammatory chemokines (MCP-1, MIP-2, IP-10, MIP-1β, KC), and reduced infiltration of neutrophils into the liver, without reducing Ad vector transduction efficacy [108].

Recently, PEGylated Ads were tested in a non-human primate model. Although not officially accepted for publication, Wonganan and Croyle suggest that when PEGylated HDAds were systemically administered in primates at the dose of 3 × 10^12^ vp/kg, reductions in several Ad-triggered toxicities (*i.e*. the lack of activation of pro-inflammatory cytokines IL-6, IL-12 and TNFα) were observed, as compared to unmodified HDAds [105]. It is not clear, however, if transduction efficiency by the PEGylated HDAds, or Ad-specific adaptive immune responses were also altered by use of the modified Ads.

In summary, Ad PEGylation approaches have made impressive progress, however, additional studies as to how PEGylation modulates the ability of modified Ad vectors to transduce host tissues (especially when utilized in primate models) are lacking. This concern is heightened as negative effects of PEGylation on Ad transduction efficiencies has also been noted in several *in vitro* and *in vivo* studies [109,110].

### 3.4. Ad targeting

Within this broad category of approaches we have included the use of specific surgical techniques, tissue specific promoters (TSP) and incorporation of receptor specific targeting ligands directly on the Ad capsid. Each of these approaches, however, attempts to maximize the transduction of specific tissues (*i.e*. liver) while simultaneously minimizing the transduction of other tissues in the host, thereby diminishing unwanted activation of the host’s immune systems.

Specific surgical, yet non-invasive injection techniques, allowing for more efficient and specific delivery of Ad vectors to the liver have been recently developed. Utilization of a catheter-based, balloon occlusion of the vena cava to facilitate increased dwell time of the Ad vector in the liver of an non-human primates, facilitated efficient long-term (over one year) transduction of liver tissues using lower amounts of the Ad vector, and significantly reduced Ad-triggered immune responses, including acute releases of systemic cytokine/chemokine release [111]. Theoretically, this method could be applied to any gene therapy protocol targeting the liver [112,113]. While we have previously noted that the expression of non-foreign transgenes from Ad based vectors can many times minimize the induction of immune responses to the transgene, the use of tissue specific or endogenous promoters to drive expression of the transgene gene can sometimes further reduce transgene specific immune responses, and facilitate long-term transgene expression from Ad based vectors as well [24,114–116]. The logic behind these strategies suggests that the duration of transgene expression in the host may be maximized, if expression of the specific transgene is prevented from occurring in off-target cells, such as APCs. Specifically, the use of an hAAT endogenous promoter in C3H/HeJ mice allowed for persistent expression of hAAT for over 10 months in mice, as compared to only 2–4 weeks of transient expression, when the ubiquitous promoter (PGK) was utilized to drive hAAT expression [115]. Similarly, expression of human apolipoprotein A-I (apo A-I) in BALB/c mice was limited due to robust humoral immune responses against the transgene when apo A-I was expressed utilizing the ubiquitous promoters derived from CMV, U1b, mMHCII or E beta; however, these transgene specific antibodies were not generated when apo A-I was expressed utilizing a hepatocyte specific promoters such as apo A-I (or hAAT, or apo CII) [114].

Modification of the Ad capsid to facilitate immune-evasion of Ad vectors has been another area of intensive investigation. These strategies parallel the logic as to the use of tissue specific promoter/enhancer elements to drive transgene expression, namely minimizing off-target side effects that may occur after Ad vector mediated gene transfer. Several Ad capsid proteins, including the fiber, penton, protein IX and hexon proteins can be utilized for genetic incorporation of foreign peptides and/or proteins, as “in-frame” insertions [16]. Numerous considerations, regarding infectivity, scalability and stability of so modified Ads should be considered and addressed, as has been described elsewhere [16,117–120].

Ad5-based vectors depend on interactions between the trimeric Ad capsid fiber protein with the cellular coxsackievirus–adenovirus receptor (CAR, member of immunoglobulin superfamily) to facilitate highly efficient levels of cell transduction. Binding of the fiber protein to CAR is followed by the binding of the Ad capsid penton proteins to cellular αvβ3 or αvβ5 integrins through a conserved Arg–Gly–Asp (RGD) consensus motif. In addition, factor X Gla domain interactions with Heparan-sulfate proteo-glycans and the hypervariable region of the Ad hexon protein were also recently identified as a critical factor mediating Ad transduction of the liver [117].

Based on this biology, modification of important fiber domains, the RGD motif within the penton, C-terminus extension of the pIX protein, or the hypervariable region of the hexon protein have been employed in attempts to modify Ad5 vector tropism. A major limitation of re-targeting approaches is that numerous promising *in vitro* results fail to provide any significant benefit *in vivo.* This is primarily due to the mammalian circulatory anatomy, and the innate immune systems, both of which have evolved to “direct” viruses (regardless of their inherent ability to bind certain cellular receptors) bacteria, and other particulate matter into the reticuloendothelial system (RES), facilitating both rapid immune recognition of potential pathogenic invaders, as well rapid clearance or destruction of the pathogens by macrophages such as the Kupffer cells in the RES of the liver and spleen [118]. However, a recent study provides optimism, as it showed that a two-fold increase in tumor uptake of a novel Ad could be achieved when that Ad vector was displaying the αvβ6 integrin-selective peptide from the Ad fiber protein. This vector was simultaneously confirmed to also have reduced levels of liver sequestration [119]. Future development of advanced targeting Ads, possibly by combining approaches undertaken to date, should be continued in order to more fully determine the applicability of de- or re-targeting Ad vectors for specific, tissue targeted clinical applications [120].

### 3.5. Ad capsid-display of immuno-evasive proteins

This approach follows on the previous strategies to display specific peptides directly from the Ad capsid. In these vectors, however, the selection of the peptides/proteins is based on their ability to inhibit or prevent the activation of important portions of the host innate immune system, in a manner that does not globally interfere with the host’s natural ability to respond to other infectious agents.

One of these portions of the innate immune response is the complement system, a system of some thirty proteins that not only rapidly interacts with and disables invading pathogens, but also acts as a central bridging mechanism facilitating adaptive immune system recognition of newly encountered pathogens. These functions are fully detailed in several review articles [121–123]. Conventional Ad vectors cause rapid complement activation *in vivo* [124], which results not only in an amplification of Ad-triggered innate immune responses, but also significantly contributes to Ad capsid and transgene dependent humoral and cellular immune responses [4–6,23]. Moreover, complement functionality results in induction of Ad capsid-specific antibody responses, including NABs [4–6,23]. Based upon these important considerations, we set out to develop a series of novel Ad5 vectors capsid-displaying specific complement inhibitory peptides or proteins [117,120]. These Ads were constructed and confirmed to have infectivity, stability and scalability similar to conventional Ads [117,120]. Two types of peptide with complement inhibitory activities were displayed: (1) a small 13 amino acid peptide (COMPinh), that had been previously shown to inhibit complement activation in human or non-human primate *in vitro* models [125]; and (2) decay accelerating factor (DAF) a natural complement inhibitor present on host cells to inactivate complement complexes deposited on the cellular surface [126]. Importantly, the COMPinh capsid-displaying Ad dramatically reduced human complement activation *in vitro* [125]. More dramatically, systemic (intravenous) injection of high doses of DAF-displaying Ads resulted in a significantly reduced induction of thrombocytopenia, endothelial cell activation, and pro-inflammatory cytokine and chemokine activations (IL-12, MCP-1) in mice, as compared to identical injection of conventional Ads [126]. Moreover, DAF-displaying Ads had a reduced ability to induce pro-inflammatory gene induction, as well as NK cell activation, as compared to conventional Ad vectors [126]. Importantly, these improvements were achieved without reducing Ad transduction efficiency, nor the need for post-production covalent modification of the vector, (thereby retaining ease of scalability inherent to Ad vector production) [126]. Future studies are now justified to more fully assess the potential of Ads capsid-displaying complement inhibitors.

### 3.6. Chimeric Ad vectors and Ad vectors, derived from alternative Ad serotypes

The “vectorization” of rare human or non-human serotypes of wild type Ads has also been extensively undertaken. These efforts primarily address several limitations to the use of conventional Ad5 based vectors, inclusive of: (1) allowing for efficient transgene expression in Ad5-pre-immune hosts, (2) potentially minimizing innate and adaptive host immune responses to the alternative serotype vector, and/or (3) to change the tissue tropism of the Ad vector. In these studies, either a sub-portion of the Ad5 vector genome is replaced with genomic elements of the alternative serotype Ad, thereby creating “chimeric” Ad vectors, or, in a more radical approach, the entire Ad vector genome is composed of proteins exclusively derived from the alternative serotype Ad [16,36,127–132].

Ad Fiber and hexon proteins have been the two main targets for genetic modifications resulting in chimeric Ads, primarily due to the fact that these two proteins are known to be major targets for neutralizing antibodies [94–98]. Numerous hexon- and fiber- chimeric Ads have been created and tested, as reviewed [16]. In general, despite the fact that not every chimeric Ad is viable [129], several significant chimeras have been designed confirmed to have an ability to evade pre-existing Ad5 immunity [16], emphasizing the importance of future studies.

Use of vectors entirely derived from “vectorization” of alternative human Ad serotypes (including Ad26 and Ad35) have also shown promising results, in particular, in terms of ability to deliver transgenes into Ad5-immune hosts [36,131,133,134]. Alternative serotype Ad vectors derived from non-human serotypes have also been developed from multiple species, including bovine, canine, chimpanzee, porcine Ads [2]. Vectors derived from Chimp Ads C1 or C68 have been tested in both rodent and non-human primate animal models [132,135]. Use of these alternative serotype based Ad vectors not only allowed for improved induction of immune responses to vector encoded antigens in vaccine applications, but use of these novel vectors also allowed for vector re-administration in Ad5-immune hosts [36,131,132]. As a result, human clinical trials utilizing Ad26 as a HIV-vaccine platform have been initiated [136,137].

The use of alternative serotype based Ads also has several important potential drawbacks. Humans have evolved in concert with exposure to human Ad viruses, and have not been exposed to Ads derived from other species. Therefore, it may be predicted that the human innate immune system may react to the capsid proteins of alternative serotype Ads in a manner that is different, and possibly more robust, than in response to the co-evolved Ad5 capsid. Recently, the innate immune response to the capsid proteins of alternative serotype Ads have not only been proven to be significantly different from the Ad5 based platforms, but these responses tend to be more vigorous, and in some instances can be quite toxic in animal models [23,36,138,139]. Alternative serotype based Ad vectors have different cell tropisms, therefore biodistribution of these vectors will be theoretically quite different than that of Ad5 vectors. The safe and widespread use of Ad5 vectors in humans over the past decade, and the knowledge gained from those experiences, cannot be fully capitalized upon when utilization of an alternative serotype Ad is proposed for a clinical application.

## 4. Conclusions and future directions

In summary, Ad-based gene transfer outcomes can be improved or expanded upon by modulation of innate and/or adaptive immune responses to the Ad vector and/or to the Ad vector expressed transgene. Several arms of the mammalian innate immune system (including the TLR and complement systems [3–6,23,51,138,140,141]) significantly mediate Ad-triggered inflammatory responses and modulate downstream adaptive immune responses to the Ad vector and/or the transgene expressed from the Ad vector. Ad activation of CD8+ T cells, CD4+ T cells and Ad specific antibody production by B cells contribute to the clearance of Ad-infected cells from the host, and may also blunt potential re-injection attempts, therefore limiting the duration and efficacy of transgene expression [142]. Therefore, specific approaches have been developed in attempts to minimize Ad-triggered toxicities, while simultaneously maximizing gene transfer efficiency.

Global modulation of the host’s immune systems are commonly used in the context of solid-organ transplantation and may promote improvements in Ad mediated gene transfer, however, these manipulations may also predispose to infections and are generally considered less optimal. Combining Ad mediated gene transfer with these regimens can and should occur in the near term, especially as more knowledge accrues in regard to the isolated use of Ad vectors. Overall, there is a great need for research studying each of the approaches described in this chapter, in non-human primate models. Risk-benefit ratios in regard to use of Ad vectors for the putative treatment of potentially lethal conditions such as urea cycle defects, muscular dystrophies (inclusive of Pompe disease) and severe cardiovascular diseases, can and will likely justify the first disease candidates that will see the use of these immuno-modulatory strategies in human trial subjects. These applications will benefit from utilization of the several approaches outlined herein, inclusive of isolated or combined use of genome modified Ad vectors, “capsid-displaying” Ads, chemically modified Ads, chimeric Ads, as well as Ad vectors derived from alternative Ad serotypes. Finally, application of these methods in the context of Ad mediated gene transfer will also support future use of these strategies in the context of non-Ad based gene transfer vectors.
